# Intestinal malrotation and Meckel’s diverticulitis in a 19-month-old boy

**DOI:** 10.1259/bjrcr.20210127

**Published:** 2021-10-28

**Authors:** Nida Mushtaq, Elliot Elwood, Esther Westwood, Alexander Macdonald, Amulya K Saxena, Josephine Bretherton

**Affiliations:** 1 Department of Radiology, Chelsea and Westminster Hospital NHS Foundation Trust, London, UK; 2 Department of Paediatric Surgery, Chelsea and Westminster Hospital NHS Foundation Trust, London, UK

## Abstract

Acute intestinal obstruction is a common paediatric surgical emergency and should be considered in any child presenting with vomiting, abdominal pain and abdominal distension. Many causes of bowel obstruction arise from congenital anomalies and recognition of the underlying cause of obstruction can be challenging in these settings. These cases can be further complicated if two or more congenital anomalies are present. Malrotation of the gut is defined as a congenital developmental anomaly of the rotation of the intestine and encompasses a spectrum of abnormalities. Meckel’s diverticulum is another congenital anomaly which occurs secondary to the failure of the vitellointestinal duct to close and can present in 2% of the population. We describe an interesting case of a 19-month-old^-^boy who presented acutely with symptoms of bowel obstruction and was found to have both intestinal malrotation and Meckel’s diverticulum.

## Clinical presentation

A 19-month-old boy presented to his local hospital with a 3-day history of non-bilious vomiting and generalised malaise. This was associated with a history of two days without opening bowels and worsening abdominal distension. His GP had started him on oral amoxicillin for a suspected tonsillitis 2 days prior to presentation whilst his mother did not report any fever or any indication that he had abdominal pain. He had been passing dark, strong-smelling urine with decreased frequency.

He was previously fit and well and had been born at term in Brazil. He was followed up by a cardiologist for left-sided superior vena cava with monthly echocardiograms until 6 months of age with no significant family history of note. He lived with his parents and 4-year-old sister, none of whom had had any vomiting.

Upon review after transfer to a specialist centre, he was clinically dehydrated with sunken eyes. His abdomen was markedly distended, but soft, non-tender and not peritonitic. He had active bowel sounds and passed a formed stool with no evidence of rectal bleeding shortly after transfer. A nasogastric tube was on free drainage with a small volume of light yellow aspirate in the bag.

## Differential diagnosis, investigations and imaging

Initial investigations showed raised inflammatory markers [white cell count (WBC) 16.5 10^9^/L (4–11 10^9^ l^−1^), C-reactive protein (CRP) 129 mg l^−1^ (<5 mg l^−1^)], and suggested dehydration [Sodium 129 mEq/L (135–145 mEq/L), urea 6.8 mmol l^−1^ (2.5–7.0 mmol l^−1^)]. On capillary blood gas, he had a slight respiratory alkalosis [pH 7.50 (7.35–7.45), pCO_2_ 42 mm Hg (35–45 mm Hg), HCO_3_ 25 mEq/L (22–28 mEq/L)], with a normal lactate (1.1).

**Figure 1.**
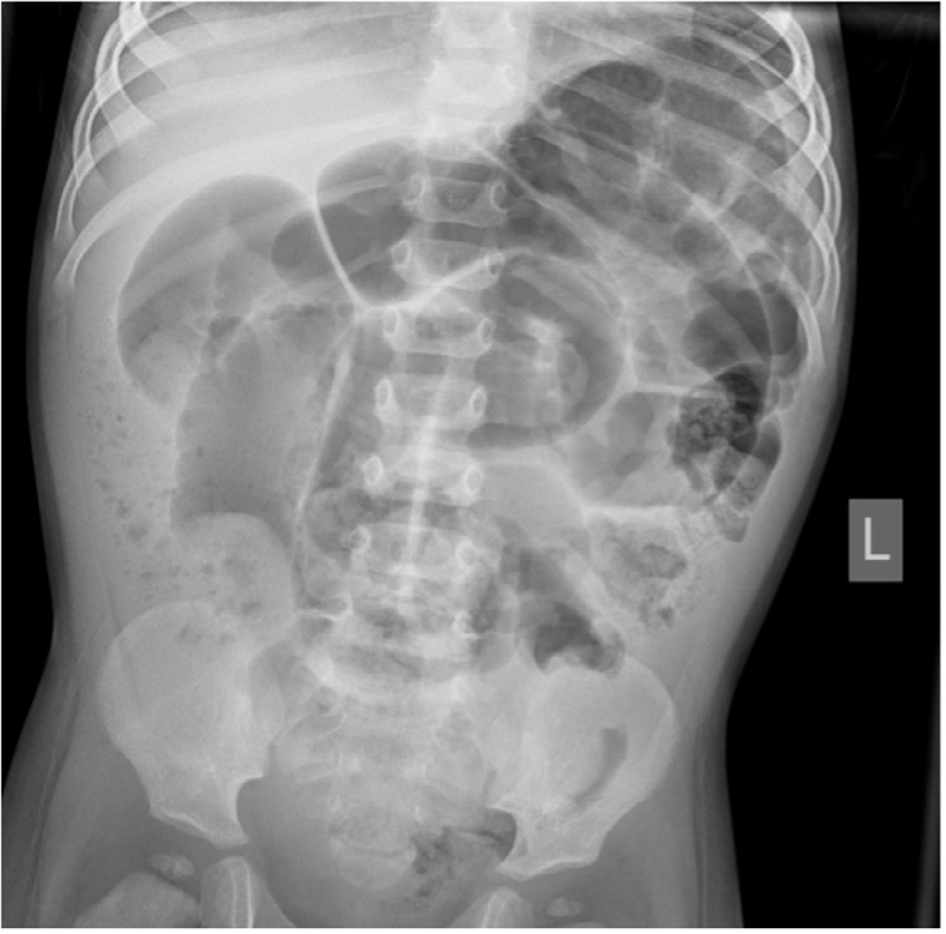
Abdominal radiograph demonstrating dilated central bowel loops with predominantly right-sided peripheral faecal loading. No evidence of pneumoperitoneum.

A plain abdominal radiograph performed at his local hospital showed multiple dilated central bowel loops with some peripheral faecal loading (Figure 1). There were no signs of pneumoperitoneum.

Upon transfer, the main differentials were intussusception or partially treated appendicitis with septic ileus. An abdominal ultrasound was performed, which demonstrated multiple dilated bowel loops and large volume free fluid. The appendix was not visualised, and no intussusception was seen.

**Figure 2.**
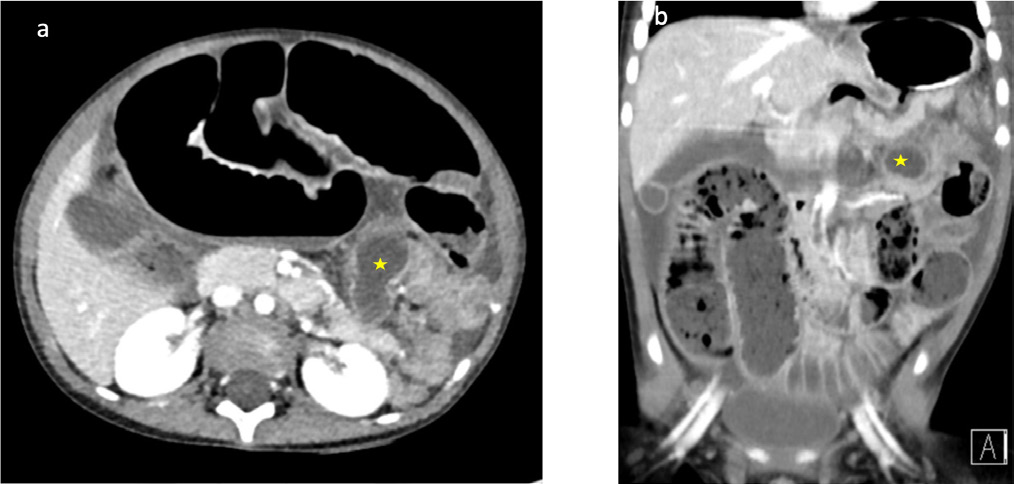
(**a, b**) Corresponding axial and coronal CT imaging demonstrating high-grade small bowel obstruction with a transition point seen leading to a dilated tubular structure measuring 18 mm seen within the left upper quadrant (yellow star)

Computed tomography (CT) abdomen and pelvis was performed for further assessment of the dilated bowel loops, and confirmed high-grade small bowel obstruction with a transition point in the left upper quadrant involving an anterolateral loop of small bowel, leading to a thin-walled, blind-ending tubular structure measuring up to 18 mm in diameter (Figure 2a and b). The appendix was not identified separately to this structure.

**Figure 3.**
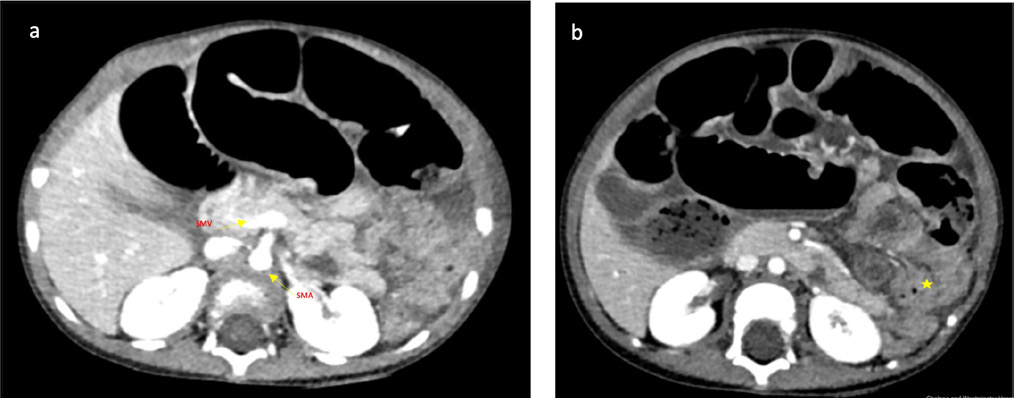
(**a, b**) Axial CT images: (**a**) The yellow arrows demonstrate the superior mesenteric vein (SMV) located slightly anterior and to the left of the superior mesenteric artery (SMA). The SMV should be positioned to the right of the superior mesenteric artery SMA. Reversal of the SMA/SMV relationship is classically associated with intestinal malrotation. (**b**) Yellow star demonstrates collapsed large bowel loops which are seen predominantly to the left of the midline, caecum could not be confidently identified.

The CT demonstrated abnormal bowel orientation with a left-sided colon, but normal appearing position of the DJ flexure (Figure 3a and b). The caecum was felt to be left-sided, but the exact location could not be confirmed. The superior mesenteric vein was located anterior and slightly to the left of the superior mesenteric artery. There was no evidence of perforation.

## Pre-operative working diagnosis

The dilated tubular structure seen on CT was felt most likely to represent an inflamed appendix in the left upper quadrant with an associated bowel obstruction, on a background of a rotational bowel anomaly given the SMA/SMV orientation and left-sided large bowel loops (Figure 3a and b). The possibility of malrotation and volvulus with a Meckel’s diverticulum was considered and discussed but felt to be less likely.

## Operative findings and treatment

**Figure 4.**
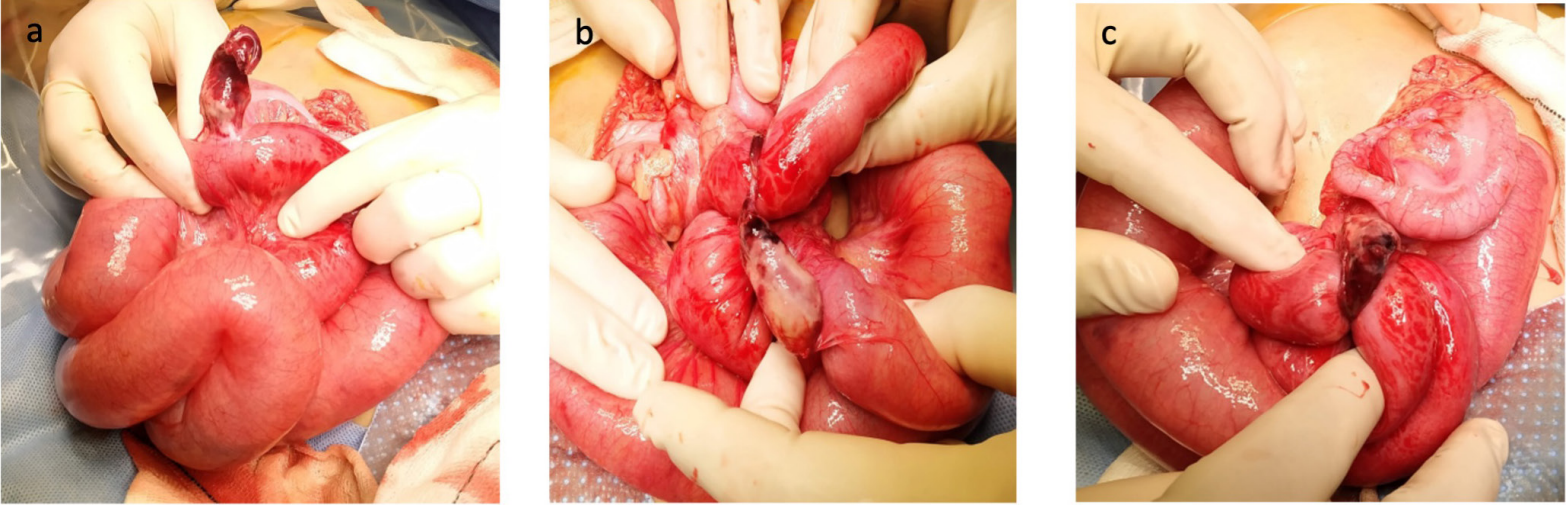
(**a–c**) Intraoperative photographs demonstrating (**a**) Meckel’s diverticulum, (**b, c**) The band arising from the diverticulum (mesodiverticular band associated with Meckel’s diverticulum) which resulted in small bowel obstruction.

The patient was subsequently taken to theatre for an exploratory laparotomy. The intraoperative findings were of a malrotation with the caecum and appendix positioned in the left upper quadrant and dense Ladd’s bands covering a right-sided DJ flexure. The terminal ileum was strangulated by a Meckel’s band (Figure 4b and c) which ended in a necrotic Meckel’s diverticulum (Figure 4a). This 1 cm of terminal ileum (2 cm proximal to the ileo-caecal valve) was ischaemic and non-viable. A Ladd’s procedure, appendicectomy and en bloc resection of the Meckel’s diverticulum and ischaemic terminal ileum with primary end-to-end anastomosis was performed.

## Outcome, follow-up and discussion

Post-operatively, the patient recovered well after a period of ileus. He had a repeat ultrasound scan after spiking a fever on the fifth post-operative day which did not show any collection, and his fevers resolved. He was discharged on the ninth post-operative day.

Malrotation is a congenital anatomical anomaly of fetal intestinal rotation. It is defined by abnormal position of the bowel within the peritoneal cavity and is usually accompanied by abnormal mesenteric bands or absence of fixation of portions of the bowel.^1,2^ There is a spectrum of appearances depending on the embryological stage of development at which the anomaly occurs, ranging from omphalocoele to non-rotation, incomplete or reversed rotation, internal hernia and mobile caecum/unattached duodenum.^3^ Malrotation can lead to the life-threatening complication of midgut volvulus, which can result in obstruction and necrosis of up to the entire midgut.^2^ The clinical incidence of malrotation is estimated to be 1 in 6000 live births and may occur as an isolated condition or in association with other anomalies. However, autopsy studies suggest the actual incidence may be as high as 1 in 100.^4^

The classical clinical manifestation of malrotation in newborns is bilious vomiting with or without abdominal distention, associated with either duodenal obstructive bands or midgut volvulus. However, in an important minority (such as this case), presentation may be atypical.^5^

Meckel’s diverticulum is recognized as the most common congenital anomaly of the gastrointestinal tract occurring in 2% of the population. It represents a remnant of the omphalomesenteric duct which connects the yolk sac to the midgut through the umbilical cord. Failure of the duct to close results in a diverticulum in 90% of cases, which can be found on the antimesenteric border of the distal small bowel, typically 100 cm from the ileocecal valve.^6^ These diverticula are typically asymptomatic however can present with complications such as diverticulitis, haemorrhage, intussusception, obstruction or neoplasm. Ectopic mucosa (most commonly gastric, found in 62%, but occasionally pancreatic or a combination) may be found on histological examination. On CT, Meckel’s diverticulum is typically seen as a blind-ending fluid filled structure in continuity with the antimesenteric border of the terminal ileum.^6^

Concurrent intestinal malrotation with Meckel’s diverticulum has been reported in a very small number of cases to date; however, it is notable that this case is unique in that the patient remained asymptomatic until this acute presentation. Prior reports of concurrent malrotation and Meckel’s diverticulum describe a more acute on chronic picture with the chronic symptoms likely secondary to episodes of subacute volvulus.^7^ These prior reports have also attributed the delayed presentation of malrotation to the absence of fibrous bands between the caecum and peritoneum.^7,8^ However, in this case, Ladd’s bands were found overlying the DJ flexure at the time of surgery and therefore it is surprising that this did not present in the neonatal period.

Intraoperatively, this patient was also found to have a mesodiverticular band which strangulated the terminal ileum and resulted in small bowel obstruction. Previous case reports of concurrent Meckel’s diverticulum and malrotation have described acute presentations secondary to malrotation with incidental intraoperative findings of Meckel’s diverticulum.^7,8^ This case is unique in that the primary cause of the acute presentation was small bowel obstruction secondary to a Meckel’s band and necrotic Meckel’s diverticulum with incidental malrotation noted both on imaging and intraoperatively.

Small bowel obstruction with a Meckel’s diverticulum accounts for 40% of symptomatic Meckel’s and can occur through a variety of mechanisms. These include trapping of a bowel loop by a mesodiverticular band (as was the case in this particular patient), volvulus of the diverticulum around a mesodiverticular band, intussusception as well as incarceration in an inguinal hernia (Littre’s hernia).^5^

Intestinal obstruction due to the mesodiverticular band of Meckel’s diverticulum is seen rarely and becomes an even more unusual occurrence in the paediatric setting. A mesodiverticular band forms due to failure of involution of one of the two embryological vitelline arteries, resulting in a fibrous band that typically extends from the tip of the Meckel’s diverticulum. This can result in bowel obstruction by strangulating a loop of bowel.^9^

In this unique case, the presence of a rotational abnormality was successfully noted and was key to highlight the possibility of pathology (such as appendicitis or Meckel’s diverticulum) usually affecting a different region of the abdomen. The pathology was localised to the left upper quadrant, which was helpful for surgical planning.

When diagnosing malrotation (on upper GI contrast studies), the typical teaching is to evaluate whether the third part of the duodenum has a retroperitoneal course and to locate the position of the DJ flexure, which in normal anatomy should be lateral to the left lumbar pedicle at the level of the gastric pylorus. On cross-sectional imaging, the SMA/SMV relationship may be reversed or abnormal in malrotation, although this is not always reliable.^10^

In this case, the third part of the duodenum did appear to have a retroperitoneal course on the CT with the DJ flexure apparently appropriately sited in the left upper quadrant. However, the CT was re-reviewed following the operative findings and in retrospect the DJ flexure position was felt to be slightly lower than the pylorus, but not convincingly right-sided on imaging. An important radiological learning point from this case is that malrotation represents a spectrum of abnormalities and the imaging findings can be subtle or indeterminate.

Another important learning point is the consideration of a Meckel’s diverticulum in addition to an inflamed appendix in the differential for a blind-ending dilated tubular structure, particularly if the structure is not definitively seen to arise from the caecal pole as in this case. Furthermore, identifying a possible malrotation was crucial to intraoperative decision-making.

In conclusion, this case of concurrent malrotation with a rare cause of small bowel obstruction presented itself with a challenging clinical and radiological scenario. This unique case highlighted the importance of considering rare causes of small bowel obstruction as well as being aware of congenital anomalies that may complicate image interpretation.

## Learning points

1. Malrotation is a spectrum of rotational abnormalities with a range of imaging appearances. Evaluate the SMA/SMV relationship and position of the DJ flexure to aid diagnosis.
2. Blind-ending tubular structure with bowel obstruction in a paediatric patient should raise the possibility of both appendicitis and Meckel’s diverticulum. Often surgery may be the only way to differentiate between these two entities.
3. Large bowel loops seen predominantly on the left and small bowel loops of the right should raise the suspicion of malrotation.
4. CT imaging may not provide a conclusive diagnosis in complex case such as this but can be helpful for localising pathology and planning surgery.
